# Chronic Eosinophilic Pneumonia in an Airbrush Painter With Poor Response to Systemic Steroids

**DOI:** 10.1177/2324709619890945

**Published:** 2019-12-01

**Authors:** Ahmed Taha, Roaa Ahmed, Nooraldin Merza, Ravindra Bharadwaj, Thien Vo, Manish Patel

**Affiliations:** 1 Deaconess Hospital, Evansville, IN, USA; 2 Ahfad University for Women, Omdurman, Sudan; 3 Texas Tech University Health Sciences Center, Amarillo, TX, USA

**Keywords:** chronic eosinophilic pneumonia, airbrush paint, organizing pneumonia, acrylic paints, street artists

## Abstract

Airbrush paints contain low-molecular-weight chemicals that can cause occupational asthma, respiratory sensitization, and hypersensitivity pneumonitis; however, its relationship to chronic eosinophilic pneumonia (CEP) has never been reported. In this article, we are presenting a unique association between CEP and prolonged exposure to acrylic airbrush paints. Unlike the vast majority of CEP patients who exhibit an excellent response to systemic steroids, our patient did not respond to systemic steroids. We believe that his prolonged exposure to airbrush paints and the evolution of organizing pneumonia might have contributed to the unsatisfactory response to systemic steroids, prolonged hypoxia, and the overall worse prognosis. There are no current data that correlate acrylic paints to the development of CEP; our report is the first to introduce a probe to further investigate this association.

## Introduction

Chronic eosinophilic pneumonia (CEP) is a rare eosinophilic lung disease of unknown etiology that is characterized and marked by alveolar filling with a mixed inflammatory infiltrate, primarily eosinophils.^1^ There are sparse data on the relationship between occupational and environmental exposures to CEP. In a few reports, an association with isocyanates and flour was established.^2^ Additionally, airbrush paints contain low-molecular-weight chemicals that are known to cause occupational asthma, respiratory sensitization, and hypersensitivity pneumonitis; however, its relationship to CEP has never been reported. This report highlights, for the first time in literature, the important association of CEP and airbrush paints. It also highlights the possible poor response to corticosteroids if organizing pneumonia evolves.

## Case Report

A 48-year-old male with medical history of major depression disorder, type 2 diabetes mellitus, and diabetic neuropathy presented with exertional dyspnea and cough productive of clear sputum for 4 weeks. He also had pleuritic chest pain, low-grade fever, chills, malaise, and 10 lbs weight loss over the same period of time. He had 12 pack-year smoking history and quit 2 years ago but denied any alcohol or recreational drug use. No history of contact with sick individuals or recent travel. His home medications were amitriptyline, duloxetine, gabapentin, and insulin. He works as a street artist using acrylic airbrush paints for almost 20 years. He was using personal and respiratory protective equipment when handling spray paint materials such as protective goggles, paint masks, and single-use gloves.

On physical examination, he was in mild respiratory distress and tachypneic, with bilateral basilar coarse crackles. His oxygen (O_2_) saturation was 86% on room air, corrected to 93% through Venti-mask O_2_ 40%. Arterial blood gas showed pH 7.46, pCO_2_ (partial pressure of carbon dioxide) 28 mm Hg, and pO_2_ (partial pressure of oxygen) 64 mm Hg. White blood cell count was 11 000 with 5% eosinophils, absolute eosinophils count 550/mL, elevated C-reactive protein, and procalcitonin was negative. On the subsequent day of admission, his white blood cell count trended up to 17 000, with absolute eosinophils count of 1300. Human immunodeficiency virus screening, anti-neutrophil cytoplasmic antibodies—perinuclear pattern, antinuclear antibody, lactate dehydrogenase, mycoplasma antibodies legionella urine antigens, *Strongyloides* immunoglobulin G, and serum *Aspergillus* antigen were all negative.

**Figure 1.**
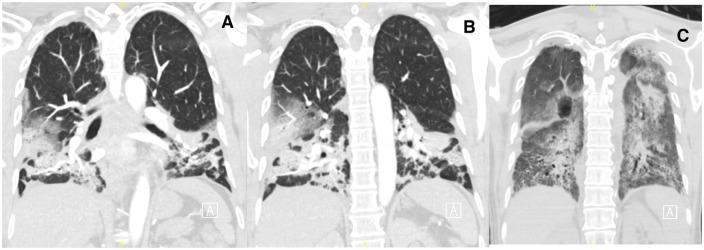
Computed tomography scan of chest at initial presentation (A and B), and after 5 weeks (C). Images A and B demonstrate bilateral airspace disease, predominantly in lower lobes, with patchy areas of ground-glass densities in a peripheral, peribronchial, and basilar distribution that was seen at the time of presentation. Image C, taken 5 weeks after presentation, shows diffuse bilateral interstitial infiltrates and interval worsening of ground-glass opacities scattered throughout both lungs. Fibrotic changes were evolving bilaterally with more dense consolidative changes in the lung bases and an interval worsening in aeration of the upper lobes.

**Figure 2.**
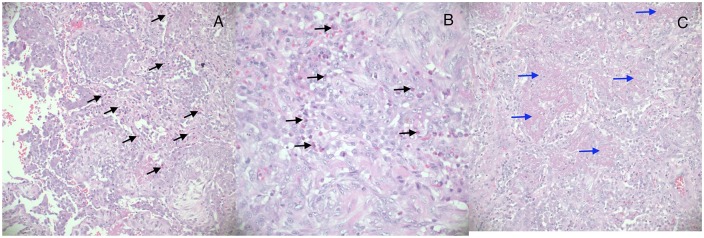
Hematoxylin and eosin section of right wedge lung biopsy. (A) Low-magnification power (×100) and (B) high-magnification power (×200), showing mixed alveolar inflammatory infiltrate with significant eosinophilic-predominance (black arrows). (C) Low-magnification field (×100) that demonstrates multinodular organizing pneumonia with fibroblastic proliferation and subpleural dense fibrous deposition (blue arrows).

Computed tomography scan of the chest at time of presentation showed bilateral airspace disease, predominantly in lower lobes, with patchy areas of ground-glass densities in a peripheral, peribronchial, and basilar distribution (Figure 1A and B). He was then started on 1 mg/kg/day of methylprednisolone due to the high suspicion of eosinophilic pneumonia. However, his respiratory distress worsened, and his oxygen requirement continued to increase, that is, he was placed on high-flow oxygen through Vapotherm. Due to the patient’s respiratory distress and high oxygen requirements, bronchoscopy was considered a high-risk procedure; therefore, open lung biopsy through video-assisted thoracotomy was obtained for a definitive diagnosis. Pathological examination of right wedge lung biopsy revealed mixed alveolar inflammatory infiltrate with significant eosinophilic-predominance, multinodular fibroblastic proliferation, and subpleural dense fibrous deposition (Figure 2), a pattern consistent with CEP and evolving organizing pneumonia.

The diagnostic thoracotomy was complicated by left apical pneumothorax requiring chest tube placement. Despite of the administration of 2 rounds of high-dose steroids, that is, 1 mg/kg/day methylprednisolone for 2 weeks followed by pulse steroid dose of 1 g/day methylprednisolone for 5 more days, the patient continued to have severe respiratory distress requiring non-rebreather mask, high-flow nasal cannula, and, at times, noninvasive positive pressure ventilation. Then, he developed respiratory muscle fatigue and hypoxic hypercapnic respiratory failure requiring endotracheal intubation. He stayed on the ventilator for 2 more weeks and received another round of pulse steroid dose with no significant improvement. A repeat computed tomography chest scan, done 5 weeks later, showed diffuse bilateral interstitial infiltrates, interval worsening of ground-glass opacities scattered throughout both lungs, and evolving fibrotic changes bilaterally (Figure 1C).

Unfortunately, adequate clinical recovery was never achieved during the hospital course and the patient remained in moderate respiratory distress with high oxygen requirements, that is, 4 L O_2_ via nasal cannula or Venti-mask 35% interchangeably. The patient then opted for a palliative level of care and was discharged to a hospice unit where he passed away 3 weeks after discharge.

## Discussion

Chronic eosinophilic pneumonia is listed as an orphan disease by the US Food and Drug Administration, defined as a condition that affects fewer than 200 000 people nationwide,^3,4^ with a reported prevalence of 0% to 2.5% among several interstitial lung diseases registries.^5^ It typically affects patients in their third or fourth decade of life and is twice as frequent in women as in men.^6^ The diagnosis is based on the association of respiratory symptoms for 2 or more weeks; alveolar eosinophilia (≥25%); blood eosinophilia (≥1000/µL); pulmonary infiltrates with peripheral predominance on chest imaging; and, more important, exclusion of other known etiologies of eosinophilic pulmonary disease.^5^ Blood eosinophilia and radiological findings are considered supporting findings—not a set of diagnostic criteria—for CEP.

Significant peripheral eosinophilia in excess of 1000/µL is reported in 88% to 95% of CEP cases.^7^ However, several cases of CEP without blood eosinophilia have also been reported.^8^ In the case series by Jederlinic et al, 19 patients with CEP were followed for 11-year period, and 37% of patients did not have significant peripheral eosinophilia.^9^ Therefore, in the absence of blood eosinophilia, cytological or histological evidence of alveolar eosinophilia in an otherwise unexplained pulmonary infiltrates represents an acceptable feature for the diagnosis of CEP.^1^ Additionally, the finding of bilateral peripheral or pleural-based, nonsegmental, consolidative opacities described as the “photographic negative” of pulmonary edema is highly suggestive of CEP when seen on chest imaging. This typical “photographic negative” pattern, however, is present in only one fourth of patients.^10^ It is also not specific for CEP, and has been described in cryptogenic organizing pneumonia, sarcoidosis, or drug-induced pneumonia.^10,11^ Additional imaging features such as ground-glass opacities and nodules (like in our case), upper lung opacities, or migratory opacities could also be seen in CEP.^7^

Although pathological confirmation of CEP through lung biopsy is the gold standard, it is typically a last resort due to its invasiveness. Lung biopsies are rarely done if the clinical diagnosis is challenging or the bronchoscopy is contraindicated or inconclusive, that is, absent eosinophilia in BAL (bronchoalveolar lavage), despite high suspicion index,^1^ as in our case. The transbronchial lung biopsy approach is not commonly performed because the small size of the specimen usually prevents making a definitive diagnosis; hence, the surgical approach is the preferred method.^7^ The histopathology usually shows an interstitial and alveolar inflammation with predominance of eosinophils. Because our patient did not have marked peripheral eosinophilia, did not demonstrate dramatic response to corticosteroids, and was not stable enough to undergo bronchoscopy, open lung biopsy was performed based on the high clinical suspicion of CEP.

A myriad of conditions can mimic the clinical presentation of CEP and often have similar laboratory findings. These include vasculitic syndromes such as Churg-Strauss syndrome (CSS), allergic reactions to fungi or helminths such as allergic bronchopulmonary aspergillosis (ABPA) or Löeffler syndrome, atypical infections like tuberculosis, or—very rarely—malignancy (eg, lung cancer or lymphomas).^5^ History, physical examination, and chest imaging in our case were not suggestive of tuberculosis or any underlying malignancy as possible alternative diagnoses. Central bronchiectasis, high total immunoglobulin E levels, positive immediate and late skin tests that are typically present in ABPA were lacking.

Churg-Strauss syndrome, a systemic eosinophilic vasculitis, typically has a long history of atopic disease including asthma and, most often, allergic rhinitis. These symptoms were absent in our patient. Extrapulmonary manifestations of CSS caused by tissue eosinophilic infiltration were also nonexistent in our patient. Pulmonary infiltrates, similar to those described in CEP, are observed in about 50% of CSS cases, but our patient did not have any of the other features that could support the diagnosis of CSS. Also, the diagnosis of Löffler syndrome, caused by the transpulmonary passage of helminth larvae, was unlikely in our case. The patient did not have relevant epidemiologic exposure to eggs of *Ascaris lumbricoides, Hookworms*, or *Strongyloides*, which is a cornerstone in the diagnosis of Löffler syndrome^12^ (no prior *Ascaris* exposure and no recent history of travel to a tropical region, where helminthic infections are prevalent). Characteristic radiographic findings of Löffler syndrome (migratory bilateral round infiltrates) were also lacking in our patient.

As with most orphan diseases, CEP can be difficult to diagnose and manage because symptoms may be confused with other more common diseases. Additionally, no sufficient data in the literature exist to support a causal relationship to a particular etiology, as the rarity of the condition makes it almost impossible to conduct epidemiological or clinical studies that are large enough to have sufficient power in order to establish causation. Several reports are available correlating possible environmental or occupational factors to the development of CEP, such as flour^13^ and isocyanates,^2^ but never to airbrush paints.

Airbrush painting works by passing a stream of compressed air through a venturi pump creating suction that allows paint to be pulled from an interconnected reservoir at normal atmospheric pressure. The high-velocity air atomizes the pigment particles into tiny droplets that range in size between 0.5 and 10 µm, depending on paint viscosity and dispersibility, the deriving pressure, and the brush resistance.^14^ Chemicals may be inhaled if the mask is poorly sealed or if the vapor penetrates through the mask because the aerosol droplets are smaller than its openings.^15,16^ Standardized protection ranges for face masks vary between 95% and 99% and could prevent penetration of particles as small as 0.4 µm.^15^ However, most self-employed street painters do not pursue a fit test before using a particular respirator nor do they genuinely seek to comply with the Occupational Safety and Health Administration recommendations regarding the most appropriate respirator type. Instead, they choose inexpensive masks that are available over the counter. This behavior further contributes to decreasing the protection threshold of the mask.^17^ Therefore, it is imperative to note that respiratory protective equipment are never 100% effective.

Acrylic-based paints are the most common form used by street artists, such as our patient, for airbrush painting.^18^ Acrylic paints consist of a pigment that gives it color, a synthetic resin binder that holds the particles of pigment together, a solvent, and other additives. Paint ingredients and pigment proportions differ per manufacturer but most contain cadmium sulfide, cobalt powder, carbon charcoal, titanium dioxide, different iron oxides, and others.^19^ Paints used in airbrushing contain low-molecular-weight chemicals that can cause occupational asthma, respiratory sensitization, and hypersensitivity pneumonitis.^15^ However, there are no current data that correlate these ingredients to the development of CEP; our report is the first to introduce a probe to further investigate this association.

Treatment of CEP is based on corticosteroids. After initiation of treatment, symptoms, as well as eosinophilia (blood and alveolar), typically regress within hours to days. Chest imaging findings also improve, but could lag up to weeks.^5^ This dramatic response to steroid therapy has been considered by several specialists as a therapeutic trial that helps in establishing the diagnosis of CEP.^1^ However, there is no consensus on the doses or the duration of corticosteroid therapy. Our patient received intravenous methylprednisolone at 1 mg/kg/day for 14 days with no remarkable clinical response recorded. Then, another course of corticosteroid therapy was introduced at a pulse dose of 1 g/day for 5 days, but no sensible clinical improvement was appreciated. The patient continued to be in respiratory distress and his oxygen requirements remained high.

The available literature and current data of CEP reports suggest that irreversible fibrosis and death secondary to CEP, as in our patient, is extremely unusual. The poor response to systemic steroids is another striking feature in our case. We believe that the prolonged exposure to airbrush paints combined with the superimposing pathology of organizing pneumonia might have significantly contributed to the unsatisfactory response to systemic steroids, the prolonged hypoxia, and the overall worse prognosis.

## Conclusions

Our report highlights a unique association between CEP and prolonged exposure to airbrush paints. We introduced a probe to further investigate the relationship between CEP and airbrush painting as an occupational or environmental hazard. Additionally, our report highlighted that prolonged exposure to the inciting agent, the evolution of organizing pneumonia, and the development of progressive fibrosis could significantly contribute to a poor response to systemic steroids in CEP and could increase disease morbidity and mortality.
